# Cubic and hexagonal liquid crystals as drug carriers for the transdermal delivery of triptolide

**DOI:** 10.1080/10717544.2019.1602796

**Published:** 2019-05-12

**Authors:** Qian-Qian Shan, Xiao-Jing Jiang, Fang-Yuan Wang, Zi-Xuan Shu, Shuang-Ying Gui

**Affiliations:** a Department of Pharmacy, Anhui University of Chinese Medicine, Hefei, China;; b Institute of Pharmaceutics, Anhui Academy of Chinese Medicine, Hefei, China;; c Engineering Technology Research Center for Modern Pharmaceutics of Anhui Province, Hefei, China

**Keywords:** Triptolide, cubic liquid crystal, hexagonal liquid crystal, microdialysis, rheumatoid arthritis, transdermal drug delivery system

## Abstract

The purpose of this study was to develop and evaluate triptolide-loaded cubic and hexagonal liquid crystals for transdermal drug delivery systems (TDDSs). We prepared and characterized triptolide-loaded lyotropic liquid crystals and evaluated for their percutaneous permeation properties *in vitro* and *in vivo*. We then used the adjuvant arthritic rat model and HaCaT cells to analyze the pharmacodynamics and conduct cell-stimulating studies of these liquid crystals. The optimized preparations were identified as cubic and hexagonal phase structures, respectively. Moreover, the *in vitro* percutaneous penetration studies demonstrated that compared to the homemade triptolide gel, cubic and hexagonal liquid crystals could significantly increase the percutaneous cumulative penetration of drugs within 48 h. Besides, the results of skin-blood synchronous microdialysis showed that the triptolide concentration in skin was higher than that in blood, and the cubic and hexagonal liquid crystals significantly increased the bioavailability of triptolide. Triptolide-loaded cubic and hexagonal liquid crystals presented excellent anti-arthritic effects, alleviating paw swelling and inhibiting inflammation by downregulating the levels of TNF-α and IL-1β. *In vitro* cell-stimulating studies displayed that triptolide-loaded cubic and hexagonal liquid crystals exhibited no obvious toxicity, which exhibited that triptolide-loaded cubic and hexagonal liquid crystals were remarkable biocompatibility. Collectively, triptolide-loaded cubic and hexagonal liquid crystals represented a promising candidate for rheumatoid arthritis therapy.

## Introduction

Rheumatoid arthritis (RA) is an autoimmune inflammatory disorder that is the leading cause of disability. It is characterized by sustained synovitis, progressive cartilage destruction, and osteoporosis. It has been reported that RA affects approximately 1% of the population and is associated with a large expenditure of healthcare resources (Feldmann, 2002; Malahias et al., 2012; Yang et al., 2017). Currently, the treatment principle of RA is mainly to control joint inflammation, prevent joint destruction, and promote joint repair (Scott et al., 2010; Kumar et al., 2016).

Triptolide (TP), a diterpene epoxy lactone isolated from the *Tripterygium wilfordii*, which exhibits a variety of pharmacological activities, including anti-rheumatic, analgesic, anti-inflammatory, immunosuppressive properties, and anti-tumor activity (Liu, 2011; Zhou et al., 2012; Zhu et al., 2014; Li et al., 2016). Research indicated it to be highly effective against many autoimmune and inflammatory diseases, especially RA (Fan et al., 2018; Wang et al., 2018). Up to now, a number of studies have demonstrated that triptolide has anti-RA effect. Triptolide can alleviate RA by downregulating neutrophil inflammatory functions effectively (Fan et al., 2016; Zhang et al., 2017; Huang et al., 2018). In recent years, triptolide is mainly used for oral and parenteral administration, but it is usually detected in organs rapidly after administration and excreted via urinary, fecal, and biliary routes (Gong et al., 2015). In addition, due to the extremely high toxicity of triptolide to kidney, liver and gastrointestinal tract, its clinical application is greatly limited. (Liu et al., 2010; Tan et al., 2018). Moreover, existing topical preparations have poor transdermal properties owing to their structural and physicochemical characteristics. Therefore, it is critical to improve the transdermal properties of triptolide transdermal delivery systems.

Lyotropic liquid crystals (LLCs), which is composed of amphiphilic lipids that self-assembly in solvents, has attracted extensive attention due to its good performance of drug loading and drug release (Zabara & Mezzenga, 2014; Linkevičiūtė et al., 2015; Martiel et al., 2015). LLCs are usually classified as lamellar (L_α_), reversed hexagonal (H_2_), and reversed cubic (V_2_) based on their different internal structures (Pershan, 1982; Kaasgaard & Drummond, 2006; Chen et al., 2014). In recent years, the cubic and hexagonal phases as the transdermal drug delivery system have received more attention. Their structures and chemical properties are similar to those of cell membranes, allowing drugs to penetrate the stratum corneum that facilitate the percutaneous penetration of drugs (Lopes et al., 2007; Maghraby, 2010; Marganit et al., 2012; Kadhum et al., 2016; Yu et al., 2016; Mei et al., 2018). Owing to these properties, V_2_ and H_2_ phases can be particularly used for the transdermal delivery system.

Currently, amphiphilic lipid materials commonly used to construct lyotropic liquid crystals are glycerol monooleate (GMO) (Milak & Zimmer, 2015) and phytantriol (PT) (Akbar et al., 2017). In recent years, PT is a biocompatible amphiphilic compound that exhibits excellent structural stability because of the lack of ester linkages and saturated phytoalkyl backbones, which has received widespread attention. (Muller et al., 2010; Rizwan et al., 2010; Astolfi et al., 2017). PT-water systems can form the V_2_ phase at 20 °C, with a transformation to H_2_ at a higher temperature (Barauskas & Landh, 2003; Barauskas et al., 2005). In addition, the V_2_ phase can be converted into the H_2_ phase by adding vitamin E acetate (VitEA) at 20 °C (Dong et al., 2006; 2015).

In present work, a self-emulsification technique was applied to prepare triptolide-loaded cubic and hexagonal liquid crystals. The formulations were verified by crossed polarized light microscopy (CPLM) and small-angle X-ray scattering (SAXS). A modified Franz diffusion cell method was used to assess the percutaneous penetration behavior of the formulations *in vitro*. The *in vivo* pharmacokinetic behavior of triptolide-loaded liquid crystals was evaluated by skin–blood synchronous microdialysis technique. A rat model of adjuvant arthritis (AA) was selected to study the anti-arthritis effect of the formulations. We evaluated the pharmacodynamics of AA rats, including paw swelling, arthritis index, and the histopathological examination of the rat synovial membrane and the levels of TNF-α and IL-1β in plasma and synovial fluid were measured. Human immortalized epidermal cells (HaCaT cells) were selected to investigate the skin irritation induced by triptolide-loaded liquid crystals and the safety of skin application was preliminarily evaluated.

## Materials and methods

### Materials

Triptolide (purity > 99.9%), used as a reference substance, was obtained by the National Institute for Food and Drug Control (Beijing, China). Triptolide (purity > 98%) was purchased from Shanghai Yuanye Biological Co., Ltd. (Shanghai, China). PT (purity > 95%) was supplied by Tokyo Chemical Industry Co., Ltd. (Shanghai, China). Carbitol was supplied by Huai’an Heyuan Chemical Co., Ltd. (Jiangsu, China). Vitamin E acetate (purity > 98%) was obtained by Hubei Xinmingtai Chemical Co., Ltd. (Hubei, China). Complete Freund’s adjuvant and DMSO were supplied by Shanghai Yuanye Biological Co., Ltd. (Shanghai, China). DMEM medium was obtained by Solaibao Technology Co., Ltd. (Beijing, China). Fetal bovine serum was obtained by Germany Serana Co., Ltd. (Shanghai, China). Trypsin and double antibody were obtained by Shanghai Biyuntian Biotechnology Co., Ltd. (Shanghai, China). Except for acetonitrile and methanol, which are chromatographic grades, all reagents are of analytical grade.

### Animals and cells

Sprague Dawley rats (200 ± 20 g) were provided by the Experimental Animal Center of Anhui Medical University (Anhui, China). The animal experiments in accordance with the evaluation plan of the Ethics Committee of Anhui University of Traditional Chinese Medicine.

HaCaT cells were incubated in DMEM containing 10% FBS and 1.5% streptomycin/penicillin at 37 °C with 5% CO_2_.

### Preparation of triptolide-loaded liquid crystal

#### Preparation of triptolide-loaded V_2_ phase

Formulations of the triptolide-loaded V_2_ phase (F1) contained PT (54 wt%), carbitol (6 wt%), water (40 wt%), triptolide (0.1 mg⋅g^−1^). PT was heated to 60 ± 0.5 °C. Meanwhile, the drug was dissolved in carbitol to prepare triptolide solution. After that, PT and triptolide were mixed, adding a specific amount of pre-warmed water, then vortex for 3 min. The resulting formulations were then maintained in closed vials at 25 °C to reach equilibrium for 72 h prior to examination.

#### Preparation of triptolide-loaded H_2_ phase

The triptolide-loaded H_2_ phase (F2) was prepared using PT, VitEA, and water (69.44:5.56:25, w/w/w) and triptolide (0.1 mg⋅g^−1^). The triptolide-loaded H_2_ phase was prepared in the same manner as the triptolide-loaded V_2_ phase, except for the addition of 5.56% VitEA to PT before melting.

### Characterization of triptolide-loaded liquid crystal

#### Cross-polarized light microscopy (CPLM)

The CPLM was used to examine the structure of the formulations and then analyzed under the cross-polarized light (CK-500, Caikon, China) at 20 °C.

#### Small angle X-ray scattering (SAXS)

We used the SAXS measurements to further evaluate the structures of the formulations. The experiments were carried out on SAXSess mc^2^ SAXS (Anton Paar, Graz, Austria). The test conditions were as follows: the X-ray source was CuKa radiation, with a wavelength of λ = 0.15418 nm. The voltage and anode current were 40 kV and 50 mA, respectively. Scattering intensities were plotted versus the scattering factor (q), according to the position of Bragg scattering peaks, the structure of the formulations was determined.

### In vitro percutaneous permeation study

#### In vitro percutaneous permeation

*In vitro* percutaneous permeation study was assessed by Franz diffusion cell (TK-6A, Shanghai Yukai Technology Trade Co., Ltd., Shanghai, China). The receptor compartment was filled with 20 mL physiological saline containing 50% methanol and stirred at 200 rpm at 32 ± 0.5 °C. The isolated abdominal skin of rats was fixed on Franz cells (surface area of 3.14 cm^−2^) with the stratum corneum facing the donor compartments. 1.5 g of LLC preparations and gel were smeared to the donor compartments. 1.0 mL of the receptor phase was withdrawn at 0.5, 1, 2, 3, 4, 6, 8, 10, 12, 24, and 48 h, and replaced with equivalent volume of receptor phase.

#### HPLC analysis

The triptolide concentrations were determined by HPLC (LC-20D Daojin, Japan). The chromatographic column was a C18 column (250 × 4.6 μm, 5 μm). Isocratic elution was performed using 26% acetonitrile and 74% ultrapure water (v/v). The wavelength was 219 nm. The flow rate and volume was 1.0 mL⋅min^−1^ and 20 μL, respectively.

### In vivo pharmacokinetic study

#### Microdialysis system

The microdialysis system (MD) composed of a syringe pump (CMA 400; CMA Microdialysis Stockholm, Sweden), perfusion apparatus (CMA 2.5 mL) and microcollector (CMA 470). The probes had a 15 kDa cutoff with a 0.6 mm membrane diameter (10 mm in length).

#### In vitro recovery of probes

Flow rates and concentrations were the important factors in determining the recovery rate of the probes. Then, we used the increment method and the decrement method to determine the recovery rates of the probes *in vitro* and *in vivo*.

##### The influence of flow rate on recovery of probes

The probes were placed in ethanol-physiological saline solution (30:70) containing 20 μg⋅mL^−1^ triptolide and maintained the temperature at 37 ± 0.5 °C while stirring at 200 rpm. The probes were perfused with a blank ethanol-physiological saline solution (30:70) at various flow rates (0.5, 1, 2 and 4 μL⋅min^−1^). Each group was collected in quadruplicate. By the increment method, the *in vitro* recovery rate of the probes was calculated from the following equation:

$$R_{i}=C_{\text{out}}/C_{\text{in}}\times 100\%$$

where R_i_ is the recovery of probes; C_in_ and C_out_ are the initial concentration and the concentration after dialysis, respectively.

The probes were placed in blank perfusion solution and perfused the ethanol-physiological saline solution (30:70) containing 20 μg⋅mL^−1^ triptolide at various flow rates (0.5, 1, 2, and 4 μL⋅min^−1^). Each group was collected in quadruplicate. The *in vitro* recovery rate of the probes was calculated using the decrement method with the equation as follows:

$$R_{d}=(C_{\text{in}}-C_{\text{out}})/C_{\text{in}}\times 100\%$$

where R_d_ is the recovery of probes; C_in_ and C_out_ are the initial concentration and the concentration after dialysis, respectively.

##### The influence of concentration on recovery of probes

The probes were placed in triptolide ethanol-physiological saline solution (30:70) with various concentrations (5, 10, and 20 μg⋅mL^−1^), and the blank perfusion solution was perfused at 1.0 μL⋅min^−1^. Each group was collected in quadruplicate. By the increment method, the *in vitro* recovery rate of the probes was calculated from the following equation:

$$R_{i}=C_{\text{out}}/C_{\text{in}}\times 100\%$$

where R_i_ is the recovery of probes; C_in_ and C_out_ are the initial concentration and the concentration after dialysis, respectively.

The probes were placed in blank perfusion solution and perfused with various concentrations of triptolide (5, 10, and 20 μg⋅mL^−1^) at the flow rate of 1.0 μL⋅min^−1^. Each group was collected in quadruplicate. The *in vitro* recovery rate of the probes was calculated with the decrement method equation:

$$R_{d}=(C_{\text{in}}-C_{\text{out}})/C_{\text{in}}\times 100\%$$

where R_d_ is the recovery of probes; C_in_ and C_out_ are the initial concentration and the concentration after dialysis, respectively.

#### In vivo recovery of probes

*In vivo* recovery rate of probes was determined by the anti-dialysis method. Rats were anesthetized with 20% urethane (0.6 mL⋅100 g^−1^ i.v.). Then the microdialysis probes were implanted in the subcutaneous tissue and right jugular vein with the help of guide cannulas, respectively. After a stabilization period of 1 h, the perfusion solution containing different concentrations (5, 10, and 20 μg⋅mL^−1^) of triptolide was perfused at the flow rate of 1.0 μL⋅min^−1^ to ensure that recovery rate of probes was concentration-independent. Studies with variable flow rates (0.5, 1, 2, and 4 μL⋅min^−1^) were performed to choose the best flow rate. The *in vivo* recovery rate of triptolide was determined by the decrement method formula.

#### Skin pharmacokinetic study

Fifteen male SD rats were randomly divided into three groups. The implantation of the microdialysis probes was the same as described in the preceding section. After a stabilization period of 1 h, triptolide-loaded V_2_ phase, H_2_ phase, and gel were evenly applied to the abdominal skin of rats on an area of 2.0 × 2.0 cm^2^ at a dose of 300 μg⋅kg^−1^. Samples were collected once every 40 min and collected continuously within 12 h.

#### UPLC-MS/MS analysis

Triptolide concentration in plasma and skin was analyzed by UPLC-MS/MS (ACQUITY UPLC/Xevo TQ-S, Waters, Milford, MA, USA). Samples were determined on an ACQUITY UPLC^TM^ BEH C18 column (2.1 × 150 mm, 1.7 µm; Waters) in isocratic elution mode. The mobile phase was composed of 33% acetonitrile and 67% ultrapure water (v/v). The flow rate was 0.2 mL⋅min^−1^ at a column temperature of 40 °C. Mass detection was conducted by positive electrospray ionization mode. The ionization conditions were as follows: nitrogen gas flow for desolvation 800 L⋅h^−1^; ion source temperature 150 °C; evaporation temperature 500 °C; cone gas flow rate 50 L⋅h^−1^; and voltage 3 kV. The ion pair and ion source parameters of triptolide were as follows: mass-to-charge ratios (m/z) 361.13→128.25; cone voltage (CV) 38 V; collision energy (CE) 50 V. The ion pair and ion source parameters of hydrocortisone (internal standard) were as follows: m/z 363.11→121.09; CV 28 V; and CE 24 V.

### Anti-rheumatoid arthritis efficacy of triptolide-loaded liquid crystals

#### Animal grouping and modeling

Eight normal SD rats were selected as normal group and 32 rats with adjuvant arthritis were selected as AA group. Furthermore, the rats in the AA group were equally divided into four groups, including model group, triptolide-loaded gel group, triptolide-loaded V_2_ group, and triptolide-loaded H_2_ group. Three weeks after successfully modeling AA, the formulations with 80 µg⋅kg^−1^ triptolide were gently smeared on the abdominal skin of rats once-daily for seven days.

#### Degree of paw swelling

The edema volume of each rat’s right hind paw was measured by d7, d14, d21, d23, d26, and d28 before and after inflammation, and the swelling degree of the secondary paw was determined by the plethysmometer (BHS-7C; Beijing Zhonghui Tiancheng Technology). We calculated the edema volume as:

edema volume = paw volume after injection − injection front paw volume

#### Arthritis index

Regularly observed the severity of arthritis in rats, and grade according to the degree of erythema and paw swelling by 0–4 points as follows (Meka et al., 2018): 0 = no swelling or erythema, 1 = swelling or erythema of the toe joints or toes, 2 = swelling or erythema at the toe joints and toes, 3 = swelling or erythema at the toe joints/toes/ankle joints, 4 = severe swelling or erythema behind the ankle joints. The individual scores of limbs were summed up to give maximum possible score of 8 per mouse.

#### Determination of TNF-α and IL-1β

The synovial membrane taken from the knee joint of the rats was treated with homogenized and then centrifuged for 20 min (–10 °C, 6000 rpm). Meanwhile, the plasma taken from the abdominal aorta was obtained and then centrifuged for 10 min (4 °C, 3000 rpm). The levels of TNF-α and IL-1β in the synovial fluid and plasma were determined using ELISA kits (Beijing Andi Hua-Tai Biological Technology, China).

#### Histopathological examination

After urethane anesthesia on Day 28, the secondary ankle joints were fixed, then decalcified for 2 weeks and embedded in paraffin. The sections were used for hematoxylin-eosin (H&E) staining. The light microscopy and photographed (BX51; Japan Olympus Optical Industry, Japan) was used to examine the histopathological changes.

#### In vitro cell-stimulating experiment

Given that HaCaT cell-stimulating tests are widely used to evaluate skin irritation induced by transdermal penetration enhancers and nanoparticles, we used HaCaT cells to study the cell stimulating properties of triptolide-loaded liquid crystals. HaCaT cells were seeded at an initial density of 5 × 10^4^ cells/well in 96-well plates and cultured with the blank-V_2_, blank-H_2_, triptolide-solution, triptolide-V_2_, and triptolide-H_2_ groups at various concentrations (10^−3^, 10^−4^, 10^−5^, 10^−6^, 10^−7^, 10^−8^, and 10^−9 ^mg·mL^−1^) for 24 h. Then, replaced medium by 20 μL MTT solution (5 g⋅L^−1^), using MTT assays kit to measure the cell proliferation. The absorbance (OD) of each well was measured at 490 nm, and the survival rate (%) of HaCaT cells in various concentrations was calculated as follows:

SR%=(OD_sample_−OD_blank_)/(OD_control_−OD_blank_)×100%

where SR% is the survival rate; OD_control_ and OD_sample_ are the absorbance of MTT solution-treated samples and triptolide-treated samples, respectively; OD_blank_ is the absorbance of blank medium.

## Results and discussion

### Establishment of HPLC method for triptolide

The HPLC method for evaluating the concentration of triptolide *in vitro* was validated. The regression equation of triptolide was Y = 29475 X − 6408.1 (R = 0.9997). The abscissa (X) is the concentration of triptolide, and the ordinate (Y) is the measured peak area. The calibration curves were linear over a concentration range of 0.25–15 mg·mL^−1^ for triptolide. The HPLC chromatogram is shown in Figure S1.

### Establishment of UPLC-MS/MS method for triptolide

A method of UHPLC-MS/MS for the determination of triptolide in dialysis solution of rats was established. Hydrocortisone was chosen as the internal standard. The results showed that the concentrations of triptolide in the range of 0.01032–103.2 µg·mL^−1^ showed a good linear relationship with the peak area, and the regression equation was Y = 0.0037 X + 0.0032 (R = 0.9998). The ratio (X) of triptolide to the internal standard concentration is the abscissa, and the ratio (Y) of the peak area of triptolide to the internal standard peak area is the ordinate. The UPLC-MS/MS chromatogram is shown in Figure S2.

#### Characterization of triptolide-loaded liquid crystal

##### CPLM

**Figure 1.**
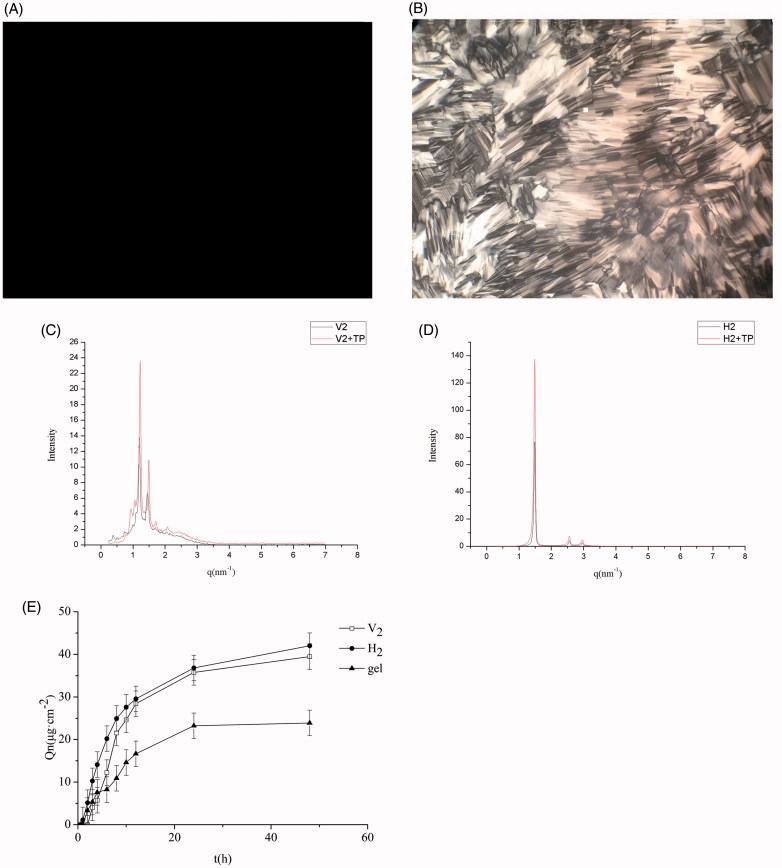
Images of triptolide-V_2_ phase (A) and triptolide-H_2_ phase (B) based on polarizing microscope (×100 magnification). SAXS profiles of triptolide-V_2_ phase (C) and triptolide-H_2_ phase (D), using blank liquid crystals as control. (E) *In vitro* permeation profiles of triptolide from V_2_, H_2_ and gel ($\bar{x}$± s, *n* = 3).

CPLM was used to characterize the structures of the formulations. The results in Figure 1(A) reveal a dark field of view, suggesting a cubic phase liquid crystal, and the results in Figure 1(B) show a fan-shaped pattern, indicating a hexagonal phase liquid crystal.

##### SAXS

We used SAXS to further confirm the liquid crystal structures of the F1 and F2 formulations, with a liquid crystal without triptolide as a control. The results display that the scattering vector ratios of the three Bragg scattering peaks are $\sqrt{2}:$ $\sqrt{3}:$ $\sqrt{4}$(Figure 1(C)) for F1 and 1:$\sqrt{3}:$ $\sqrt{4}$ (Figure (1D)) for F2. These consequences proved that F1 was cubic liquid crystal and F2 was hexagonal liquid crystal. This is consistent with previous reports (Dong & Boyd, 2011). Moreover, the addition of 0.1 mg⋅g^−1^ of triptolide had no effect on the phase structure.

#### In vitro skin permeation study

*In vitro* permeation profiles of triptolide from the V_2_, H_2_, and the gel are represented in Figure 1(E). Compared to the gel group, the V_2_ and H_2_ phase notably enhanced the percutaneous penetration of triptolide *in vitro* (*p*<.05). The cumulative penetration of triptolide from the V_2_ and H_2_ phase were 1.65 times and 1.76 times than that of the gel, respectively. This result may be explained by the fact that the V_2_ and H_2_ had a lipid layer arrangement similar to the surface of the stratum corneum that their percutaneous permeation properties were superior to gel. Compared with V_2_ group, H_2_ group exhibited a higher percutaneous penetration effect. These results are in line with those of previous studies (Lopes et al., 2007; Yamada et al., 2011). It is speculated that triptolide is a fat-soluble drug. The unique liquid crystal structure of hexagonal liquid crystal is highly effective for embedding the drug, rendering it easier to penetrate the stratum corneum to promote drug penetration.

#### In vivo pharmacokinetic study

#### In vitro recovery of microdialysis probes

We used the increment and decrement methods to examine the effect of various flow rates and concentrations on recovery of probes *in vitro*. The results in Table S1 indicate that the probes’ recovery of triptolide decreased as the flow rate increased. Table S2 displays that when the flow rate was 1 μL⋅min^−1^, the average recoveries of probes by increment and decrement methods were 37.29 ± 0.32% and 38.55 ± 0.35%. The different concentrations made no difference in the recovery of probes, which indicated that there was no correlation between probes’ recovery and surrounding drug concentration. The recoveries assessed by the increment method, which were coincided with the decrement method and it laid a foundation for the *in vivo* recovery of the anti-dialysis method.

**Table 1.** Pharmacokinetic parameters in skin ($\bar{x}$ ± s, *n* = 5).

PK parameters	Groups		
	gel	V_2_	H_2_
AUC_(0–t)_/μg·(mL·min)^−1^	790.2 ± 0.29	1501.8 ± 0.41	1062.967 ± 0.15
AUC_(0–∞)_/μg·(mL·min)^−1^	801.746 ± 0.26	1513.134 ± 0.93	1078.147 ± 0.77
MRT_(0–t)_ /min	266.646 ± 0.11	340.264 ± 0.33	278.377 ± 0.26
MRT_(0–∞)_ /min	270.588 ± 0.15	342.983 ± 0.6	281.53 ± 0.52
t_1/2z_/min	57.131 ± 0.88	54.977 ± 0.62	51.428 ± 0.5
T_max_/min	200	320	240
C_max_/μg·mL^−1^	3.55 ± 0. 32	6.61 ± 0.35	5.02 ± 0.29

**Table 2.** Pharmacokinetic parameters in blood ($\bar{x}$ ± s, *n* = 5).

PK parameters	Groups		
	gel	V_2_	H_2_
AUC_(0–t)_/μg·(mL·min)^−1^	514.6 ± 0.21	813.01 ± 0.264	977.8 ± 0.57
AUC_(0–∞)_/μg·(mL·min)^−1^	537.03 ± 0.267	818.751 ± 0.218	988.619 ± 0.97
MRT_(0–t)_ /min	302.386 ± 0.08	339.788 ± 0.1	316.302 ± 0.18
MRT_(0–∞)_ /min	319.328 ± 0.32	344.073 ± 0.25	320.693 ± 0.33
t_1/2z_/min	35.272 ± 0.44	69.315 ± 0.8	67.985 ± 0.4
T_max_/min	240	360	280
C_max_/μg·mL^−1^	1.71 ± 0.26	3.21 ± 0.9	3.91 ± 0.51

#### In vivo recovery of microdialysis probes

We used the anti-analysis method to investigate the effect of different flow rates and concentrations on the recovery of probes. The results in Table S3 exhibit that the *in vivo* recovery of probes decreases with increasing flow rate, with some differences in the recovery rates of different types of probes. The results in Table S4 show that the *in vivo* recovery rates of different concentrations were similar, which indicated the recovery rate *in vivo* was not affected by drug concentration. Thus, the *in vivo* recovery rate can be used to correct the data in the microdialysis test.

#### In vivo pharmacokinetic study

**Figure 2.**
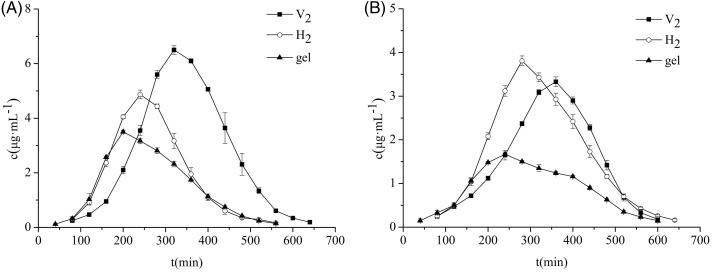
Concentration-time curves of triptolide in skin (A) and plasma (B) ($\bar{x}$ ± s, *n* = 5).

The results obtained from the pharmacokinetic study of the skin are displayed in Figure 2(A) and Table 1. It can be seen that the area under the curve [AUC _(0–∞)_] and the peak concentration (C_max_) of the V_2_ and H_2_ groups were higher than gel. The AUC(0–∞) of the V_2_ and the H_2_ groups were 1.89 times and 1.34 times that of the gel group, respectively, which indicated that the cubic and hexagonal liquid crystals could remarkably improve the percutaneous properties of triptolide that enhanced its bioavailability. The mean residence time (MRT) and peak time (T_max_) of the cubic and hexagonal liquid crystals were longer than the gel, demonstrating that the triptolide in the cubic and hexagonal liquid crystals was more concentrated in the subcutaneous tissue through the stratum corneum, which played a role in sustained release effect. The AUC, MRT, and C_max_ of the V_2_ group were higher than those of H_2_ group, which showed that the drug in the V_2_ group had a longer residence time under the skin and the concentration of the local action drug was higher than that of the H_2_ group.

The pharmacokinetic parameters of the plasma are shown in Figure 2(B) and Table 2. The AUC and MRT of the cubic and hexagonal liquid crystals were higher than those of the gel group, testifying that the triptolide-loaded in the liquid crystal was more easily permeated into the blood after transdermal administration and remained *in vivo* for a longer period of time. The C_max_ and AUC of the H_2_ group were higher than those of V_2_ group, which proved that H_2_ rendered greater absorption of the drug into the body with a systemic effect that was exerted smoothly. The T_max_ and MRT of the V_2_ group were higher than those of H_2_ group, which confirmed that the triptolide-loaded V_2_ group offered more sustained release.

Comparing the pharmacokinetic parameters of skin and plasma for each group, it was found that the C_max_ of the triptolide in skin were significantly higher than those in plasma, indicating that triptolide-loaded in cubic and hexagonal liquid crystals can reduce the side effects when it was used to treat RA. The V_2_ group had the slowest onset of action and its skin retention drug concentration was high with a strong local effect. The H_2_ group entered the blood and rendered absorption of the drug at a higher concentration, with a stronger systemic effect. Cubic and hexagonal liquid crystals exhibit different pharmacokinetic behaviors that may be attributed to their different internal structure and viscosity. Furthermore, the channel and rate of drug delivery also affect the absorption and distribution of the drug.

### Anti-rheumatoid arthritis efficacy of triptolide-loaded liquid crystals

#### Degree of paw swelling

**Figure 3.**
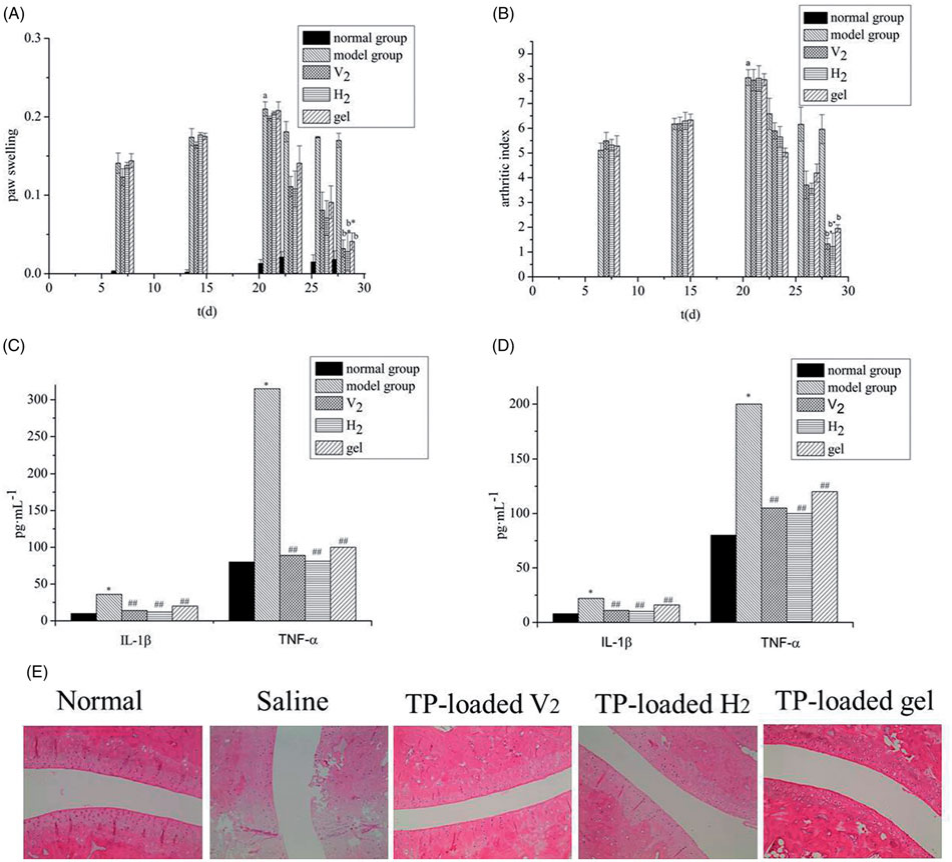
Anti-rheumatoid arthritis effects: (A) Edema volume of rats with different treatments; (B) Arthritic index scores of rats with different treatments ($\bar{x}$ ± s, *n* = 8). ^a^ *p*<.01 vs. normal group, ^b^ *p*<.01 vs. model group, *p<.05 vs. gel group. The expression of inflammatory factors in blood (C) and synovial fluid (D). ($\bar{x}$ ± s, *n* = 8). *p<.01 vs. normal group, ^##^ *p*<.01 vs. model group. (E) Photomicrographs of histopathological sections of ankle joints from rats after different treatments.

Figure 3(A) shows the changes in paw swelling in each group. After seven days of modeling, the paw swelling of AA rats gradually increased with the prolongation of the disease course. Compared with the normal group, AA rats had a significant increase in secondary lateral swelling 21 days after inflammation (*p*<.01), indicating successful modeling. The drug-administered group displayed a notably inhibited degree of paw swelling than the model group (*p*<.01) with a significant distinction between the V_2_ and the H_2_ groups compared with the gel group (*p*<.05).

#### Arthritis index

Figure 3(B) exhibits the changes in arthritis index. On day 21, the arthritis index of the model group improved remarkably compared with the normal group (*p*<.01), indicating successful modeling. The drug-administered group had an inhibitory effect on secondary joint disease in AA rats, and the arthritis index was significantly reduced. There was a significant difference between the V_2_ and H_2_ groups compared with the gel group (*p*<.05), with no significant difference between the V_2_ and the H_2_ groups.

#### Determination of TNF-α and IL-1β

Figure 3(C,D) shows the level of TNF-α and IL-1β in the plasma and synovial fluid. The rats treated with CFA overexpression of inflammatory cytokines TNF-α and IL-1β, which indicated the systemic and local inflammatory states of rats have existed. The levels of TNF-α and IL-1β in the plasma and synovial fluid of the administration groups were significantly different from those in the model group (*p*<.01), which indicated that each administration group experienced a certain inhibitory effect on both inflammatory cytokines. Moreover, the anti-inflammatory effect of the gel group was significantly lower than that of the V_2_ and H_2_ groups (*p*<.05). It was found that the hexagonal group lowered the IL-1β and TNF-α level significantly, indicating the H_2_ group experienced a substantial anti-inflammatory effect with significant effects on local and systemic inflammation. The basic pathological changes of RA include joint inflammation and cartilage destruction, both of which are closely related to the expression of inflammatory cytokines (e.g. IL-1β and TNF-α) (Matsumoto et al., 2015; Zhou et al., 2018). Current clinical treatments for RA involve TNF-α and IL-1β inhibitors to control the development of inflammation. Therefore, lyotropic liquid crystals as drug carriers offer promising therapeutic for the clinical treatment of RA.

#### Histopathological examination of the ankle joint in rat

The results of the histopathological for the ankle joint are presented in Figure 3(E). The normal group showed no neutrophil infiltration and no synovial hyperplasia; the surface of articular cartilage was smooth and without bone erosion. As expected, the model group presented a high level of inflammation with synovial hyperplasia, pannus formation, neutrophil infiltration, and a synovial lining cell proliferation. However, gel-treated AA-rats had mild inflammation with smooth articulation of the cartilage surface and neutrophil infiltration gradually eased. Compared with the gel group, the V_2_ and H_2_ groups exhibited a better anti-inflammatory effect, with no synovial hyperplasia, pannus formation, neutrophil infiltration, and a synovial lining cell proliferation. These results indicated that the transdermal administration of triptolide-loaded liquid crystals had a remarkable effect on RA.

#### In vitro cell-stimulating experiment

**Figure 4.**
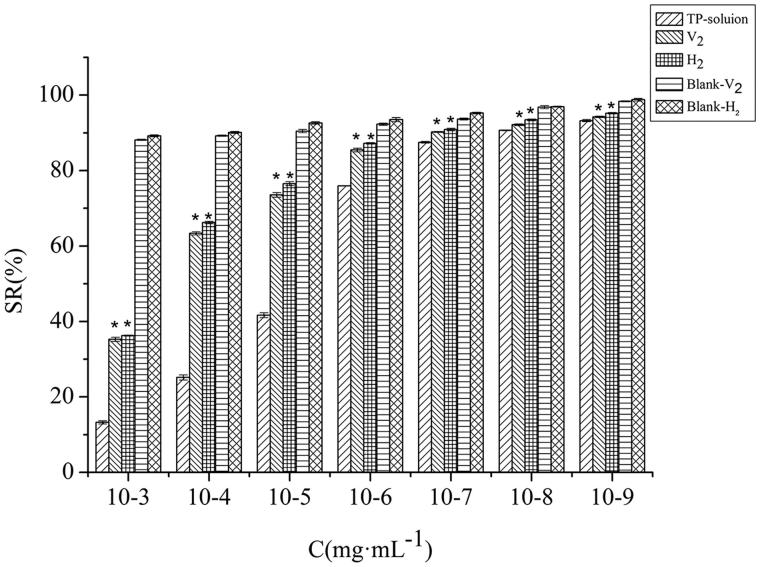
Toxicity of different treatments at different concentrations ($\bar{x}$ ± s, *n* = 6). *p<.01 vs. triptolide-solution.

We assessed the cell-stimulating of HaCaT cells treated with triptolide -solution, V_2_, H_2_, Blank-V_2_, and Blank-H_2_ for 24 h and used MTT method to measure cell viability. As can be seen from the Figure 4, the effects of different concentrations of each group on the survival rate of HaCaT cells were concentration-dependent. The V_2_ and H_2_ groups at concentration of 10^−7 ^mg·mL^−1^ triptolide has excellent biocompatibility, due to the cells presented over 90% viability. There was a significant difference in cell viability between lyotropic liquid crystal groups and the triptolide-solution group (*p*<.01). Moreover, the blank lyotropic liquid crystals displayed less cytotoxicity at the experiment concentrations. Table S5 exhibits that, the IC_50_ of the solution group, V_2_ group, and H_2_ group were 5.13 × 10^−5^, 8.13 × 10^−3^, and 1.18 × 10^−3^, respectively. These results show that lyotropic liquid crystals represent a promising transdermal delivery system with low skin irritation.

## Conclusion

In present study, triptolide-loaded cubic and hexagonal liquid crystals were prepared by self-emulsification technique using PT as matrix materials. Triptolide-loaded cubic and hexagonal liquid crystals enhanced permeation of triptolide into the skin with high bioavailability. Moreover, triptolide-loaded cubic and hexagonal liquid crystals exhibited significant anti-rheumatoid arthritis effects with biological safety. From the findings, the application of triptolide-loaded cubic and hexagonal liquid crystals as transdermal delivery vehicles offers a promising enhancement for RA therapeutics. Further research should focus on investigating the underlying mechanisms of the transdermal absorption of triptolide-loaded cubic and hexagonal liquid crystals.

## Supplementary Material

supplemental_file.doc
