# Supplementary material for: Cubic and hexagonal liquid crystals as drug carriers for the transdermal delivery of triptolide
Source: Drug Deliv. 2019 May 12;26(1):490–8. doi: 10.1080/10717544.2019.1602796 (PMC6522916; doi:10.1080/10717544.2019.1602796)
Supplement: supplemental_file.doc [file IDRD_A_1602796_SM4810.doc]

Table S1 Effect of perfusion flow rate on recovery by increment and decrement method from the microdialysis probes *in vitro*(x ± s, n = 4).

| Flow rate  (μL⋅min^-1^) | 0.5 | 1 | 2 | 4 |
| --- | --- | --- | --- | --- |
| Increment  method R (%) | 47.97±0.21 | 36.74±0.34 | 22.03±0.14 | 11.44±0.43 |
| Decrement  method R (%) | 51.07±0.65 | 37.76±0.26 | 24.43±0.77 | 12.88±0.89 |

Table S2 Effect of concentration on recovery by increment and decrement method from the microdialysis probes *in vitro* (x ± s, n = 4)

| Concentration  (μg⋅mL^-1^) | 5 | 10 | 20 |
| --- | --- | --- | --- |
| Increment  method R (%) | 37.56±0.44 | 37.43±0.31 | 36.89±0.21 |
| Decrement  method R (%) | 38.47±0.32 | 38.22±0.54 | 38.96±0.18 |

Table S3 Effect of perfusion flow rate on recovery by decrement from the microdialysis probes *in vivo* (x ± s, n = 5)

| Transdermal  administration  site | Flow rate (μL⋅min^-1^) | | | |
| --- | --- | --- | --- | --- |
|  | 0.5 | 1 | 2 | 4 |
| Skin | 50.12±0.86 | 37.23±0.54 | 23.33±0.38 | 12.44±0.47 |
| Blood | 47.34±0.27 | 34.41±0.69 | 29.69±0.71 | 13.77±0.42 |

Table S4 Effect of concentration on recovery by decrement method from the microdialysis probes *in vivo* (x ± s, n = 5).

| Transdermal  administration  site | Concentration (μg⋅mLl^-1^) | | |
| --- | --- | --- | --- |
|  | 5 | 10 | 20 |
| Skin | 35.41±0.34 | 34.92±0.56 | 35.23±0.59 |
| Blood | 37.65±0.29 | 37.42±0.75 | 36.98±0.72 |

Table S5 IC_50_for HaCaT cells with different treatments (x ± s, n = 6)

| Groups | IC_50_（mg·mL^-1^） |
| --- | --- |
| Triptolide-solution | 5.13×10^-5^ |
| V_2_ | 8.13×10^-3*^ |
| H_2_ | 1.18×10^-3*^ |

^*^*p* < .01 vs. Triptolide-solution.


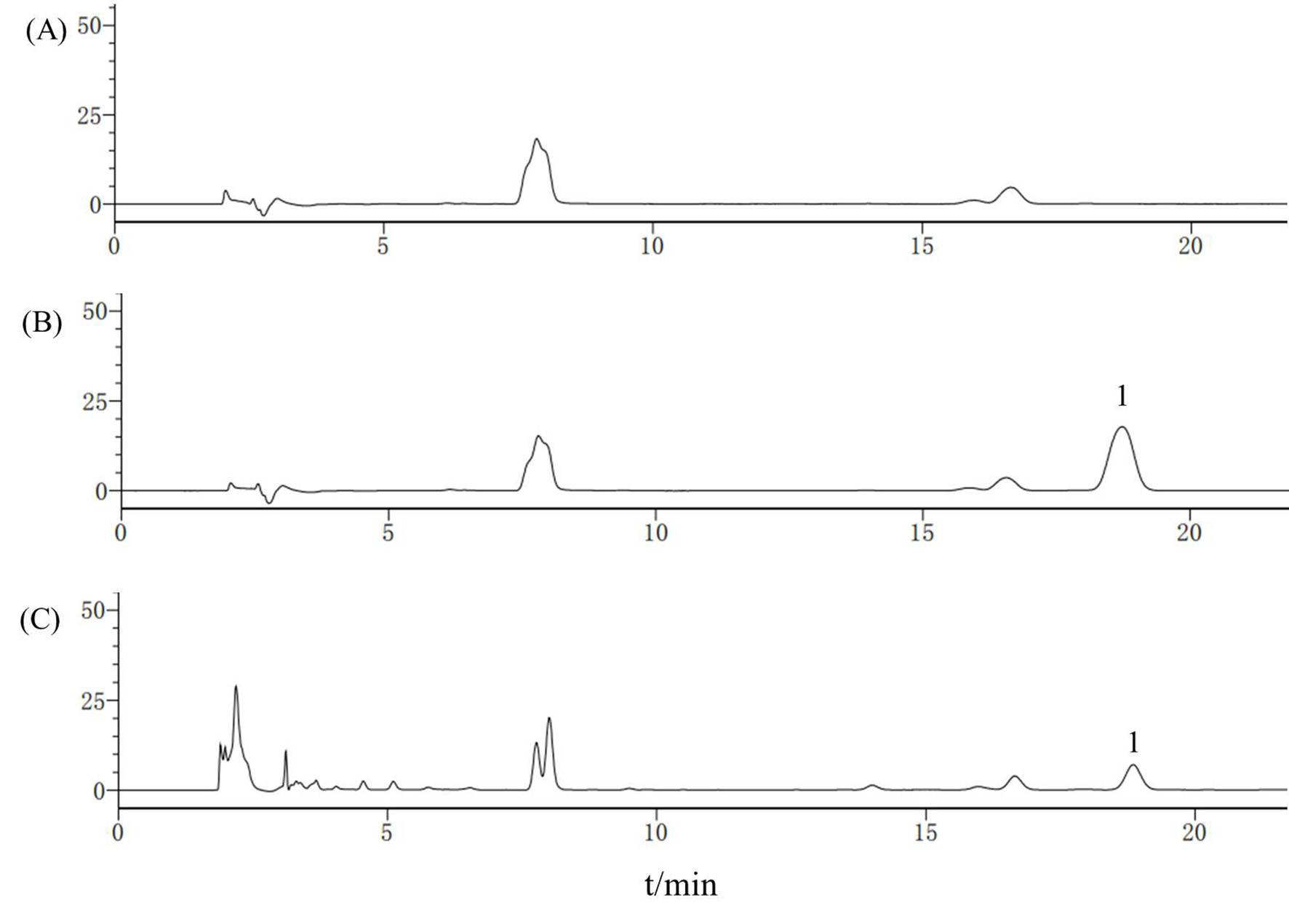


Figure S1 HPLC chromatograms of triptolide: (A) negative sample; (B) reference sample; (C) sample; 1-triptolide.


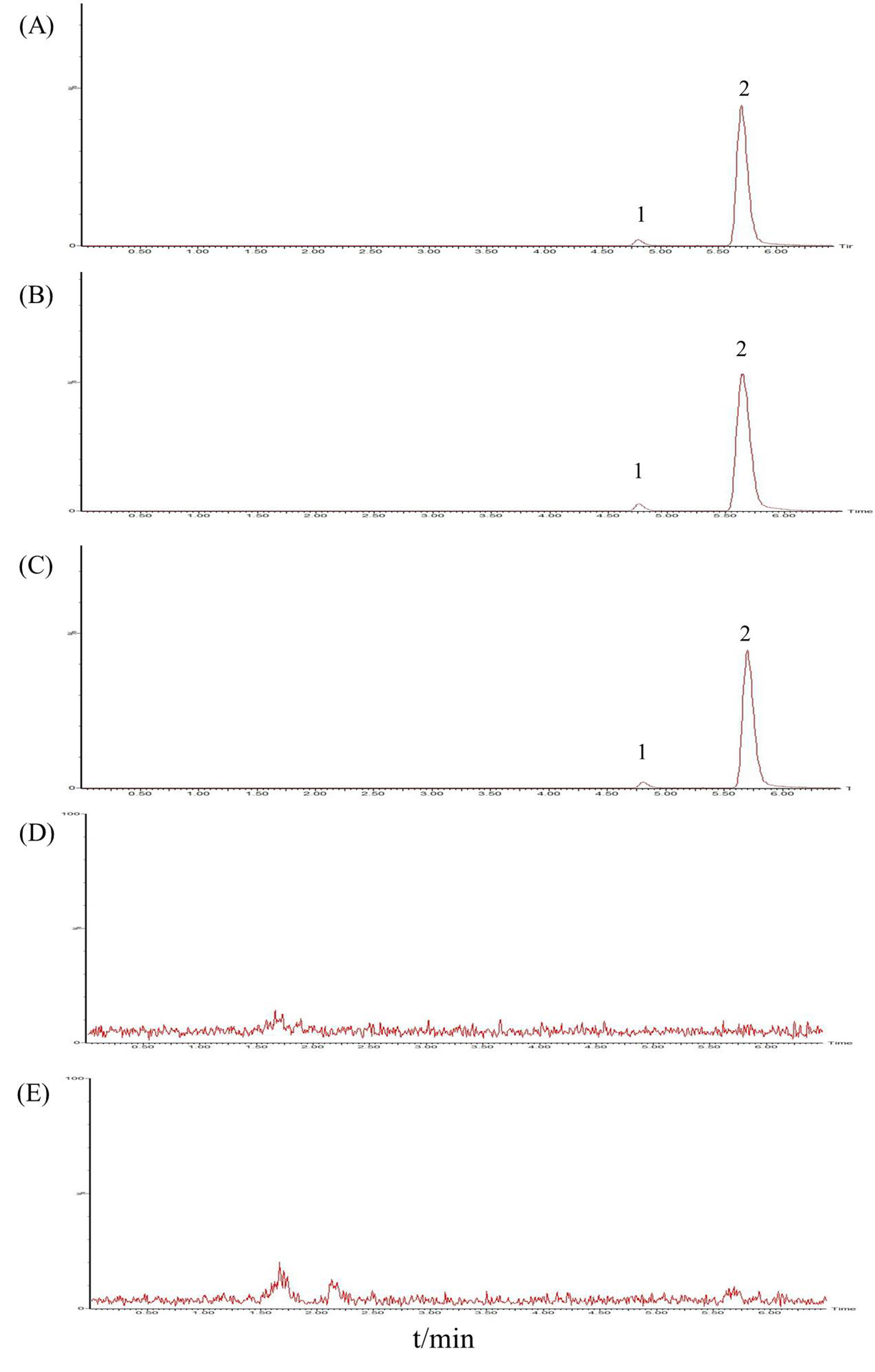


Figure S2 UPLC-MS/MS chromatograms of triptolide: (A) reference sample; (B) skin dialysate; (C) blood dialysate; (D) blank dialysate of skin; (E) blank dialysate of blood. 1: hydrocortisone; 2: triptolide.
